# Executive Functions During Submaximal Exercises in Male Athletes: Role of Blood Lactate

**DOI:** 10.3389/fpsyg.2020.537922

**Published:** 2020-10-30

**Authors:** Marinella Coco, Andrea Buscemi, Paolo Cavallari, Simona Massimino, Sergio Rinella, Marta Maria Tortorici, Tiziana Maci, Vincenzo Perciavalle, Matej Tusak, Donatella Di Corrado, Valentina Perciavalle, Agata Zappalà

**Affiliations:** ^1^Department of Biomedical and Biotechnological Sciences, University of Catania, Catania, Italy; ^2^Study Center of Italian Osteopathy, Catania and Horus Social Cooperative, Catania, Italy; ^3^Department of Pathophysiology and Transplantation, Faculty of Medicine and Surgery, University of Milan, Milan, Italy; ^4^Alzheimer’s and Psychogeriatrics Center, Mental Health Department, ASP, Catania, Italy; ^5^Faculty of Human and Society Sciences, Kore University of Enna, Enna, Italy; ^6^Department of Social and Humanistic Sciences in Sport at Faculty of Sport, University of Ljubljana, Ljubljana, Slovenia; ^7^Department of Education Sciences, University of Catania, Catania, Italy

**Keywords:** submaximal exercise, blood lactate, executive function, man, sport

## Abstract

The present study was carried out among 20 healthy young male athletes to determine whether aerobic exercise performed at two different intensities is able to affect executive functions. For this purpose, we used the Stroop Color Word Test (SCWT) to evaluate the ability to inhibit cognitive interference and the Trail Making Test (TMT) to assess organized visual search, set shifting, and cognitive flexibility. Simple Reaction Time (RT), as a measure of perception and response execution, was also evaluated. The experimental protocol included the measure of blood lactate levels with the aim of assessing possible relations between lactate blood values and selected executive functions after a 30-min steady-state test performed at 60% and at 80% of VO_2max_. The results showed that a 30-min aerobic exercise is not associated with a worsening of executive functions as long as the blood lactate levels stay within the 4 mmol/l threshold.

## Introduction

It has been observed that acute but submaximal exercise facilitates inhibitory control (e.g., Hillman et al., 2003; Drollette et al., 2014), working memory (e.g., Coles and Tomporowski, 2008; Pontifex et al., 2009), and cognitive flexibility (Bae and Masaki, 2019). These abilities fall within those cognitive capabilities called executive functions (Diamond, 2013), a term used to designate the set of cognitive abilities that allow us to plan, control, and regulate behaviors advantageous to accomplishing a certain result (Miller and Cohen, 2001).

On the contrary, some data lead to the conclusion that exhaustive exercise has the opposite effect on some executive functions and that this negative influence seems to be related to the increase of blood lactate levels (Coco et al., 2009, 2016, 2020; Perciavalle et al., 2015, 2016a,b; Coco et al., 2018; Itagi et al., 2018; Moreira et al., 2018).

Moreover, studies related to attentional processes (Coco et al., 2009, 2019b; Pellerone et al., 2017; Petralia et al., 2018; Santisi et al., 2018) have observed the negative effects of high blood lactate levels on cognition, even when induced through an intravenous infusion of lactate solution.

However, it has been observed (Perciavalle et al., 2014) that the negative effects of acute exercise on attentional processes only occurred when the intensity of the exercise exceeded 80% of maximal oxygen consumption (VO_2max_). We therefore wanted to verify whether it is possible to detect negative effects of aerobic exercise on executive functions with an intensity of more than 80% of the VO_2max_ and the concentration of blood lactate above 4 mmol/l. This value is a standard marker, called Onset of Blood Lactate Accumulation (OBLA), defined as the point when blood lactate starts to accumulate, and represents the transition from aerobic to anaerobic work (see Faude et al., 2009).

Therefore, we performed a study on healthy young athletes to determine whether submaximal aerobic exercise, carried out at two different intensities, is capable of influencing executive functions. For this purpose, we used the Stroop Color Word Test (SCWT) to evaluate the ability to inhibit cognitive interference (Strauss et al., 2006) and the Trail Making Test (TMT) to assess organized visual search, set shifting, and cognitive flexibility (Tombaugh, 2004). Simple Reaction Time (RT), a measure of perception and response execution (Woods et al., 2015), was also evaluated.

Our working hypothesis was that the possible influences exerted by aerobic activity on executive functions were linked to an increase in blood lactate values. For this purpose, the experimental protocol included the measure of blood lactate levels with the aim of assessing possible relations between lactate blood values and the selected executive functions after a 30-min steady-state test performed at 60% and at 80% of VO_2max_.

## Materials and Methods

### Participants and Procedure

**FIGURE 1:**
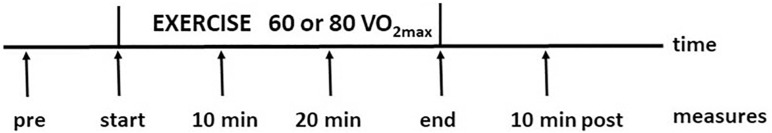
Schematic representation of the experimental protocol. Blood lactate levels were quantified before the exercise (pre), every 10 min during exercise (during), immediately at the end of exercise (end), and finally, 10 min after exercise was completed (post). The Simple Reaction Time, Stroop Color Word Test, and Trial Making Test were measured immediately at the end of the exercise (end) and 10 min after the exercise was completed (post).

The study was made possible by the voluntary participation of 20 male athletes from the Track and Field Team of the University. They had a mean age of 23.9 years (±2.25 Standard Deviations, SDs), a mean height of 125.8 cm (±4.09 SDs), a mean body mass of 74.1 kg (±4.49 SDs), a mean Body Mass Index of 24.8 (±0.79 SD) and a mean VO_2max_ of 63.9 ml/kg/min (±2.92 SDs). The protocol of the study was approved by the Ethical Committee of the University of Milan (number 15/16). All tests were performed under close clinical supervision. The athletes were fully informed about the purpose of the study and the possible risks before signing the informed consent form, in accordance with the ethical standards laid down in 1964 by the Declaration of Helsinki. Figure 1 summarizes the experimental protocol.

### Measure

#### Exercises

The experimental protocol required each athlete to perform two different aerobic exercises, with an interval of 1 week apart from each other, respectively, at 60% VO_2max_ and at 80% VO_2max_, randomly selected. Each athlete was instructed not to perform aerobic activities in the 24 h preceding the experimental session and to fast for 3 h before the exercise. The exercise, to be performed between 9 and 12 a.m., consisted of pedaling for 30 min on a cycle-ergometer (Ergomedic 828E, Monark, Sweden) at 60 or 80% VO_2max_, at a constant pedaling rate of 60 rpm (Perciavalle et al., 2015). Each athlete initially cycled without load for 3 min; then the load was increased by 30 Watt every 3 min until 60 or 80% of VO_2max_ was reached.

#### VO_2max_

VO_2max_ of each subject was calculated before the experimental sessions as previously described (Perciavalle et al., 2014). The subjects were asked to pedal continuously on the cycle ergometer throughout the experimental session, with the workload being progressively increased every 3 min for an overall duration of 30 min. Achievement of VO_2max_, defined as the highest value of VO_2_ reached during the exercise, was confirmed by the following criteria: (1) exceeding 90% of the maximum heart rate expected for the age of the athlete and (2) plateau of VO_2_ (cfr. Howley et al., 1995). Metabolic parameters were collected and assessed by using an open circuit spirometer Fitmade MED (Cosmed s.r.l. Italy), and heart rate was measured with a heart rate monitor Polar (Gays Mills, Wisconsin, United States).

#### Blood Lactate

Blood lactate levels were quantified before the exercise (pre), every 10 min during the exercise (during), immediately at the end of the exercise (end), and finally, 10 min after the exercise was completed (post). Lactate measurements were taken using a “Lactate Pro 2” portable lactate analyzer (Arkray Inc, Japan), which has proven to be highly reliable (Buckley et al., 2003).

#### Simple Reaction Time

The method for quantifying RT, a task that demands an intense simple attention, was the same one previously used (Coco et al., 2009). The volunteer was asked to press, as quickly as possible, the space bar on a computer keyboard when the “star” symbol appeared on the screen. To avoid habituation, the frequency of the appearance of the “star” symbol was randomly changed between 1 and 3 s. The evaluation was carried out immediately at the end of the exercise (end) and 10 min after the exercise was completed (post).

#### Stroop Color Word Test

The golden version of the SCWT was used in the present study (Strauss et al., 2006). The test consisted of three successive moments. Initially, the volunteer had to read a list of 50 names written in black ink. Subsequently, the subject had to indicate the color of 50 circles painted with different colors. Finally, the subject was given a list of 50 colors dyed with a color that was different from what the name indicated and asked to indicate the color of each word. The number of correct answers the subjects gave within the first 45 s of the third test represented the “interference” factor of the SCWT. The evaluation was carried out immediately at the end of the exercise (end) and 10 min after the exercise was completed (post).

#### Trial Making Test

TMT was chosen to assess organized visual search, set shifting, and cognitive flexibility (Tombaugh, 2004). The TMT is typically designed in two parts. In TMT-A the subject must use a continuous line to connect with 25 numbered circles in numerical order, distributed on a sheet of paper. In TMT-B the subject must use a line to connect 25 circles that carry letters and numbers, distributed on a sheet of paper, alternately following alphabetical and numerical order. In each of the two tests the time required for completion and the number of errors were measured.

The B/A ratio of performance on the TMT was also measured since it provides an index of executive function (Arbuthnott and Frank, 2000).

The evaluation was carried out immediately at the end of the exercise (end) and 10 min after the exercise was completed (post). The order of cognitive tests was randomly selected.

Participants were familiarized with the cognitive test procedures before the actual experiment starts in order to minimize practice effects (Theisen et al., 1998; Oberste et al., 2019), during the VO_2max_ testing day. The aim was to allow the subject to complete the cognitive tests in less than 10 min.

#### Statistical Analysis

After data collection, the mean value (±SD) of each measured parameter was calculated. Data were compared with one-way repeated measures analysis of variance (ANOVA; Friedman test), followed by Tukey’s Multiple Comparison Test. The presence of possible correlations between parameters was verified by means of two-tailed Pearson’s correlation. Significance was established at *p* < 0.05. The analyses were carried out by using GraphPad Prism version 6.03 for Windows (GraphPad Software, San Diego, CA).

## Results

**FIGURE 2:**
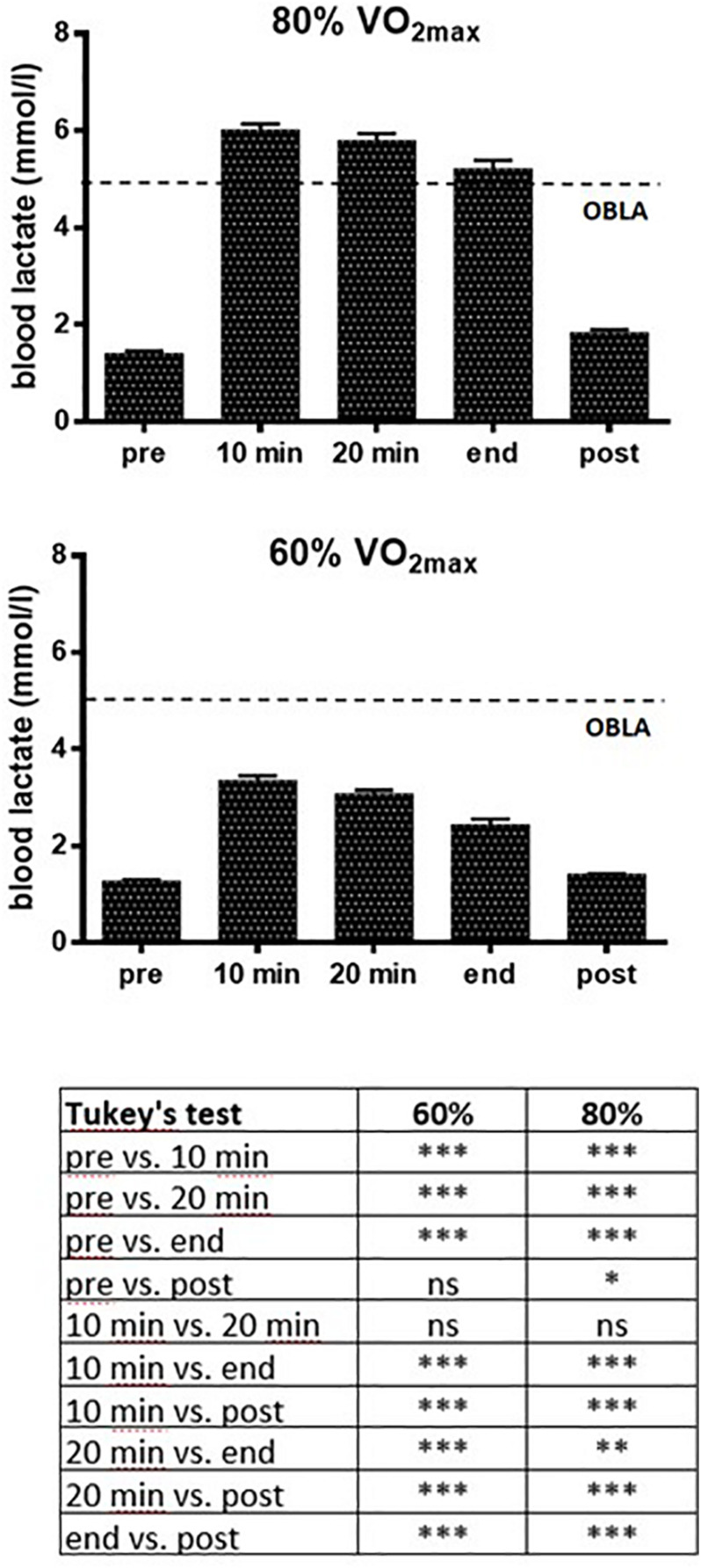
Blood lactate levels (mean values ± SD) of the 20 participants performing a voluntary 30-min exercise at 60 and 80% VO_2max_. Measurements were carried out every 10 min before the exercise (pre), during the 30-min exercise, and 10 min after the conclusion (post). The dotted line indicates the level (4 mmol/l) of the Onset of Blood Lactate Accumulation (OBLA). Results of Tukey’s Multiple Comparison Test carried out on data are also presented. ANOVA with Tukey’s multiple comparison test are the following: ^∗^*p* < 0.05, ^∗∗^*p* < 0.01, ^∗∗∗^*p* < 0.001.

Figure 2 shows the mean values of blood lactate measured in the 20 athletes in the two different motor tasks (60 and 80% VO_2max_). Blood lactate levels were quantified before the exercise (pre), every 10 min during the exercise, immediately at the end of the exercise (end), and finally, 10 min after the exercise was completed (post).

One-way ANOVA, followed by Tukey’s Multiple Comparison Test, performed on data displayed in Figure 2, shows that at both 60 and 80% of VO_2max_ the levels of blood lactate exhibits a significant (*p* < 0.001) increase, compared to the pre-exercise (pre) and post-exercise (post) values, immediately after the beginning of the exercise. During the exercise at 60% of VO_2max_ the blood level of lactate reached a maximal mean value of 3.35 mmol/l (±0.97 SD), whereas during exercise at 80% of VO_2max_ the blood level of lactate reached a maximal mean value of 6.02 mmol/l (±0.52 SD), thus overcoming the OBLA (4 mmol/l). At 80% of VO_2max_ the level of blood lactate remained over the OBLA throughout exercise, and the blood lactate returned to pre-exercise values after 10 min from its end.

**FIGURE 3:**
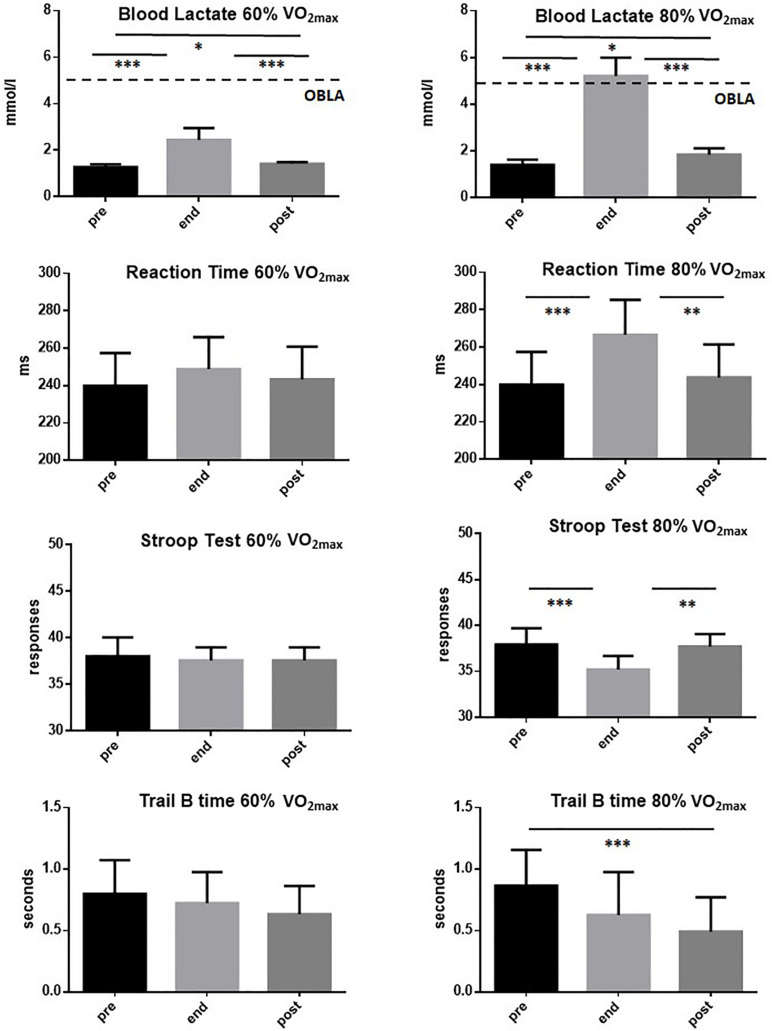
Mean values (±SD) of blood lactate, reaction time, Stroop test, and time for execution of Trial Making Test B of the 20 participants performing a voluntary 30-min exercise at 60 and 80% VO_2max_. Measurements were taken 10 min before (pre) the beginning of exercise (gray area), at its end and 10 min after the conclusion (post). The dotted line indicates the level (4 mmol/l) of OBLA. Symbols from ANOVA with Tukey’s multiple comparison test: ^∗^*p* < 0.05, ^∗∗^*p* < 0.01, ^∗∗∗^*p* < 0.001.

With respect to executive functions, as can be seen in Figure 3, it was found that while exercise performed at 60% of VO_2max_ does not change any of the parameters studied, exercise performed at 80% of VO_2max_ negatively affects RT, SCWT, and the time for execution of TMT B. No effect has been detected at either 60 or 80% of VO_2max_ on TMT-A and on the number of errors in TMT-B (data not shown). It is important to note that exercise performed at 60% of VO_2max_ caused only minimal increases in blood lactate levels, while the exercise performed at 80% of VO_2max_ caused an increase in blood lactate values above 4 mmol/l.

**FIGURE 4:**
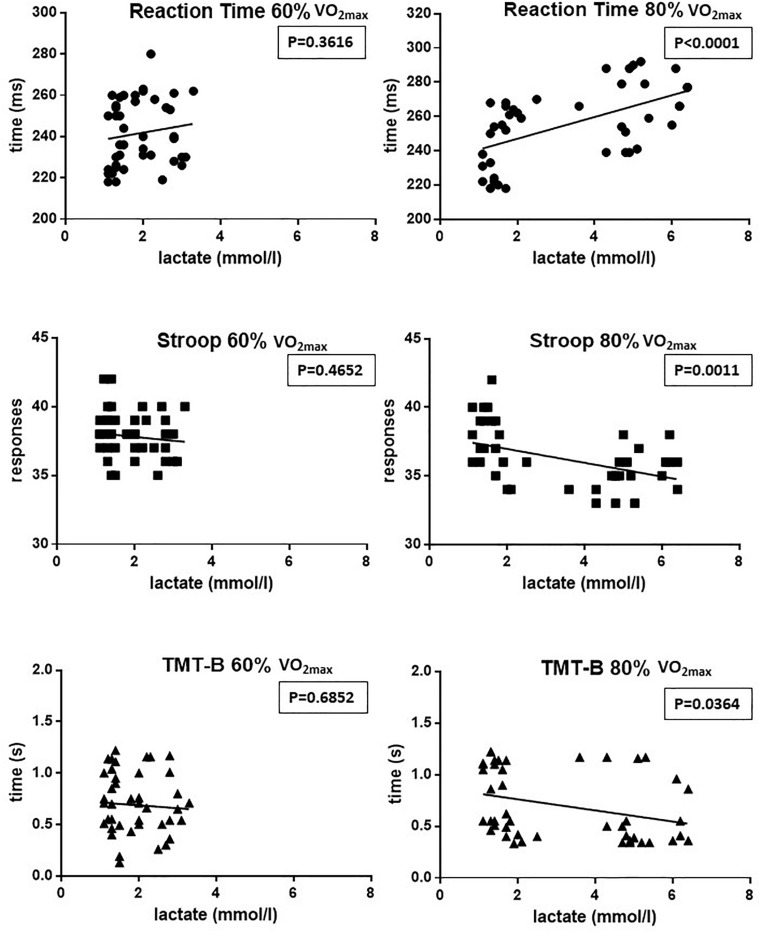
Correlations between blood lactate levels and performance measured for RT, SCWT, and TMT-B in the two different exercise intensities (60% of VO_2max_ on the left and 80% of VO_2max_ on the right). As can be seen, while at 60% of VO_2max_ none of the correlations reaches statistical significance, at 80% of VO_2max_ the 3 correlations are statistically significant (*P* > 0.05).

Figure 4 illustrates the correlations between blood lactate levels and performance measured for RT, SCWT, and TMT-B at the two different exercise intensities (60% on the left and 80% on the right).

First, it is possible to detect that during the exercise performed at 60% of VO_2max_ no correlation was found between the blood lactate levels and the measured values for RT, SCWT and TMT-B. On the other hand, it is possible to detect in Figure 4 that, during the exercise performed at 80% of VO_2max_, there is a significant positive correlation of blood lactate levels with RT and a significant negative correlation of lactate with both SCWT and TMT-B performance.

## Discussion

The present study has shown that, during aerobic exercise for 30 min, a significant worsening of the executive functions can be detected only if the intensity of the exercise is more than 80% of VO_2max_. In these conditions it was possible to detect that blood lactate levels were above the OBLA, i.e., 4 mmol/l, for the entire duration of the exercise. The present observation is consistent with what was previously reported, namely, that only aerobic activities exceeding 80% of VO_2max_ are associated with increases in blood lactate above OBLA (e.g., Chicharro et al., 1999).

The result is also in agreement with the observation that only aerobic exercise performed at an intensity that exceeds OBLA is associated with a worsening of attentive processes (Perciavalle et al., 2014). Finally, during exhaustive exercise, a worsening of working memory (Perciavalle et al., 2015) and of some executive functions (Coco et al., 2020) has been observed. Therefore, the deterioration of executive functions, observed throughout submaximal aerobic exercise carried out at 80% of VO_2max_ can be likely related to increased blood lactate levels.

It has been previously reported (Coco et al., 2009) that, after exhaustive exercise, the significant increase in blood lactate levels is associated with a worsening of attention processes. In the same study, it was also found that a lactate infusion in subjects who did not perform any physical activity is associated with a significant worsening of attentive capabilities.

It is interesting to note that it has recently been found that an acute sprint interval exercise is able to improve cognitive functions 20 min following exercise (Kujach et al., 2020). Because it is well-known that blood lactate levels return to pre-exercise levels within 15 min after the end of the performance (cfr. Coco et al., 2009), the positive effects on cognition can hardly be attributed to lactate.

This has allowed the authors to conclude that high levels of blood lactate are capable *per se* of determining a negative effect on the attentive abilities. The present study reinforces the idea that the increase in blood lactate is linearly correlated with a worsening in the efficiency of cognitive processes. The circulating lactate seems to be able to determine these negative effects both when it increases after exhaustive exercise but also when it increases during aerobic activity.

The possibility that the effects of high intensity exercise on cognitive processes may also depend on other factors (metabolic, vascular, or thermic, etc.) cannot be excluded. However, since lactate receptors have been found in the brain (Morland et al., 2015), a role for lactate as a neural regulator could be proposed (see Proia et al., 2016; Coco et al., 2019a).

One limitation of the present study is that it was conducted on young athletes, so it is our intention in the future to test the behavior of a group of sedentary subjects. Another potential limitation is the fact that the study was conducted on only 20 subjects who, although fairly homogeneous in age, gender, and degree of training, constitute a rather limited sample.

## Data Availability Statement

The datasets generated for this study are available on request to the corresponding author.

## Ethics Statement

The studies involving human participants were reviewed and approved by the Ethical Committee of the University of Milan (number 15/16). The patients/participants provided their written informed consent to participate in this study.

## Author Contributions

MC, ViP, AB, DD, MT, AZ, and VaP contributed to the conception and design of the study. MC, AB, DD, and ViP were responsible for data collection and statistical analysis. MC, AB, AZ, and ViP were responsible for the drafting and finalization of the manuscript. All authors contributed to manuscript revision and approved the submitted version.

## Conflict of Interest

The authors declare that the research was conducted in the absence of any commercial or financial relationships that could be construed as a potential conflict of interest.
